# A New Optical Configuration for the Surface Encoder with an Expanded *Z*-Directional Measuring Range

**DOI:** 10.3390/s22083010

**Published:** 2022-04-14

**Authors:** Yifan Hong, Ryo Sato, Yuki Shimizu, Hiraku Matsukuma, Wei Gao

**Affiliations:** 1 Precision Nanometrology Laboratory, Department of Finemechanics, Tohoku University, Sendai 980-8579, Japan; hong.yifan.p7@dc.tohoku.ac.jp (Y.H.); ryo.sato.t5@dc.tohoku.ac.jp (R.S.); hiraku.matsukuma.d3@tohoku.ac.jp (H.M.); i.ko.c2@tohoku.ac.jp (W.G.); 2 Division of Mechanical and Space Engineering, Graduate School of Engineering, Hokkaido University, Sapporo 060-8628, Japan

**Keywords:** surface encoder, scale grating, reference grating, Fizeau-type measurement method, *Z*-directional measuring range

## Abstract

This paper proposes a new optical configuration for a two-axis surface encoder that can measure the in-plane (*X*-axis) and out-of-plane (*Z*-axis) displacements of a positioning stage. The two-axis surface encoder is composed of a scale grating and a sensor head. A transparent grating is employed in the sensor head for measurement of the *Z*-directional displacement of the scale grating based on the Fizeau-type measurement method; a reference beam reflected from the transparent grating and the zeroth-order diffracted beam from the scale grating are superimposed to generate an interference signal. A pair of prisms and a beam splitter are also employed in the sensor head, so that the positive and negative first-order diffracted beams can be superimposed over a long working distance to generate an interference signal for measurement of the *X*-directional displacement of the scale grating. Focusing on the new, extended *Z*-directional measurement mechanism, proof-of-principle experiments were carried out to verify the feasibility of the proposed optical configuration for the surface encoder that can measure the uni-directional displacements of a scale grating along the *X*- and *Z*-axis. Experimental results from the developed optical configuration demonstrated the achievement of a *Z*-directional measuring range of ±1.5 mm.

## 1. Introduction

Machine tools and measuring instruments are required to be improved for the miniaturization of semiconductor devices and for the improvement in the fabrication accuracy of optical elements. Precision stage systems are often employed in such machine tools and measuring instruments for precision positioning [1,2,3,4]. A nanometric resolution, as well as high throughput capability, is necessary for such a positioning stage used in ultra-precision applications [5,6,7]. In the closed-loop control of a positioning stage, the accuracy and resolution of the positioning are greatly influenced by the performance of displacement sensors employed in the positioning system [8,9,10,11]. Therefore, such displacement sensors are expected to have a high resolution, as well as a large measuring range.

Laser interferometers and linear encoders are widely used for the measurement of the position of a linear stage [12,13,14,15]. A laser interferometer is vulnerable to environmental factors since its optical path is proportional to its measuring range. For high-precision measurement, it is thus necessary to strictly control the air pressure, temperature and humidity [16,17,18]. In contrast, the optical path of a linear encoder is proportional to the distance between its scale grating surface and its sensor head, which is quite small compared to that of a laser interferometer, and thus a linear encoder is expected to be more stable under environmental disturbances [19]. However, due to the limited working distance of a linear encoder, a laser interferometer-based measuring system is often employed for the measurement of multi-axis displacement of a positioning stage [20,21,22]. In such a measurement system, consisting of multiple interferometers, it is sometimes difficult to align the motion axis of a positioning stage with the measurement axis of a displacement sensor due to restrictions emanating from the mechanical structure of the system; this results in a measurement error called Abbe error [20,23]. In the case where the distance between the motion axis of the stage and the measurement axis of the displacement sensor is long, the reading of the displacement sensor could be seriously affected by Abbe error. This is a problem that cannot be ignored when carrying out nanometric measurement of stage displacement. In addition, multi-axis laser interferometer-based measuring systems tend to be large and complicated.

A better solution for measurement of a multi-axis stage system is to employ a surface encoder [24,25,26]. The surface encoder consists of an *XY* scale grating and a sensor head. The sensor head is equipped with an *XY* reference grating which has a grating pattern identical to that of the scale grating. Superposing the diffracted laser beams from these two gratings can generate interference signals that can be employed to measure the multi-axis displacement of the scale grating. The entire system can thus be simplified while also reducing the size of the optical sensor head. In addition, Abbe error can be avoided because there is only one measurement point [20,23]. A three-degrees-of-freedom (DOF) surface encoder capable of measuring *X*- and *Y*-directional in-plane displacements and *Z*-directional out-of-plane displacement has already been developed [24,25]. Furthermore, a six-DOF surface encoder has also been proposed [26] by combining a three-DOF displacement surface encoder and a three-DOF laser autocollimator [27] in such a way that they share the same measurement laser beam. Meanwhile, in these surface encoders, the measurement principle of the *Z*-directional displacement is based on the interference of the first-order diffracted beams from the scale grating and the reference grating; namely, it is based on the Michelson-type measurement method, whose non-common optical path is relatively longer than that of a common path measurement method such as Fizeau-type measurement methods [28,29]. A longer non-common optical path means that the sensor is more sensitive to environmental disturbances. Moreover, the *Z*-directional measuring range of the conventional surface encoder is limited to several hundred micrometers due to the nature of its optical design; when the scale grating moves along the *Z*-direction, the first-order diffracted beams shift with respect to the reference beam, making it difficult to overlap them with each other, and interference signals cannot be generated. Therefore, it is difficult to employ a conventional surface encoder for the measurement of a stage system whose *Z*-directional travel range exceeds the tiny *Z*-directional measuring range of the surface encoders.

In response to the background described above, in this paper, a Fizeau-type optical configuration for the surface encoder with an expanded out-of-plane *Z*-directional measuring range was newly designed and constructed. The surface encoder satisfied the measurement of the *Z*-directional out-of-plane displacement of a stage with a large measuring range. We describe the basic principle of the newly developed surface encoder, and the experiments carried out to verify the feasibility of the proposed method. It should be noted that the experiments described in this paper are for proof-of-principle, focusing on the new, extended Z-measurement mechanism in the proposed surface encoder. For the sake of simplicity, the surface encoder was designed to carry out measurement of uni-directional displacement of a scale grating in the *Z*- and *X*-directions by employing fewer optical components.

## 2. Design of the Two-Axis Surface Encoder

**Figure 1:**
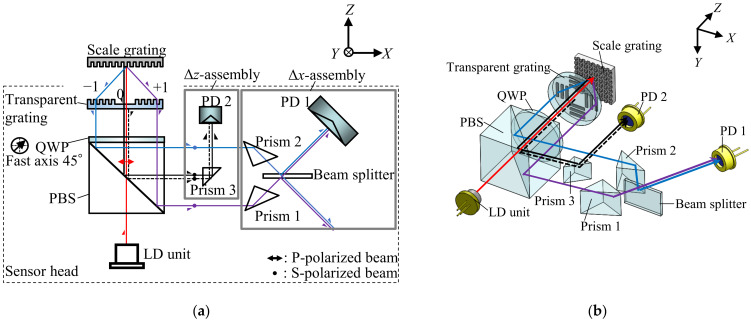
Optical design of the surface encoder: (**a**) *XZ* view; (**b**) three-dimensional view.

Figure 1 shows the optical design of the newly proposed surface encoder. It is composed of a sensor head and a scale grating. It uses a laser diode (LD, HL6750MG, Thorlabs, Newton, NJ, USA) with a wavelength of 685 nm as the light source. The polarization state of the laser beam is adjusted to P-polarization with respect to a polarizing beam splitter (PBS, PBS-20-6700, Sigmakoki, Japan). The laser beam passes through the PBS and is transferred into a circularly polarized beam after passing through a quarter-wave plate (QWP, WPW-20C-4M-45/70, Sigmakoki, Japan), whose fast axis is set to 45 degrees with respect to the polarization direction of the incident laser beam. A transparent grating, with a line grating pattern with a period identical to that of the scale grating (1 μm), is employed in the sensor head. The transparent grating is fabricated using the same system as the scale grating, except it is not coated with chromium and reflective aluminum layers [25,30,31,32]. A part of the laser beam is reflected from the surface of the transparent grating, while the rest is projected onto the scale grating. The propagation directions of the first-order diffracted beams from the scale grating are aligned to be parallel with the zeroth-order diffracted beam, using the transparent grating. The polarization states of the diffracted beams, after passing through the QWP, become S-polarization, and then the diffracted beams are reflected by the PBS. Then, the diffracted beams enter a Δ*x*-assembly and a Δ*z*-assembly for measurement of the *X*- and *Z*-directional displacements Δ*x* and Δ*z* of the scale grating, respectively.

**Figure 2:**
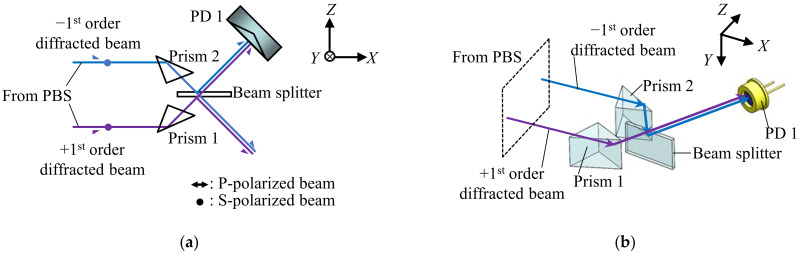
The optical design of Δ*x*-assembly: (**a**) *XZ* view; (**b**) three-dimensional view.

Figure 2a shows the schematic of the Δ*x*-assembly extracted from Figure 1a, and Figure 2b shows its three-dimensional view. As can be seen in the figures, the positive and negative first-order diffracted beams are reflected at Prisms 1 and 2 (RPB2-10-550, Sigmakoki, Japan), respectively, to be superimposed on a beam splitter (BS, model: BSS10R, Thorlabs). A photodiode (PD1, S1336-44BQ, Hamamatsu Photonics, Shizuoka, Japan) is prepared to observe the interference signal generated as the consequence of superimposing the first-order diffracted beams.

The electric fields of the positive and negative first-order diffracted beams *E*_±1_ can be expressed by the following equation:

(1) $$E_{\pm 1}=A_{\pm 1}\exp\left(i\varphi_{\pm 1}\right)$$

where *A*_±1_ are the complex amplitudes, and *φ*_±1_ are the phase shifts that can be calculated as follows:

(2) $$\varphi_{\pm 1}=\pm 2\pi \frac{\Delta x}{g}+2\pi \frac{\left(1+\cos\theta \right)\Delta z}{\lambda }$$

where *g, λ, θ* are the period of the scale grating, the light wavelength of the laser and the diffraction angle of the first-order diffracted beams, respectively. Δ*x* can be obtained by the interference signal generated by the positive and negative first-order diffracted beams. The electric field of the interference signal can be obtained as follows:

(3) $$E_{x}=E_{+1}+E_{-1}$$

Consequently, the intensity of the interference signal *I_x_* observed by PD 1 can be calculated as the conjugated complex multiplication of *E_x_* as follows:

(4) $$I_{x}=\frac{1}{2}E_{x}\cdot \bar{E_{x}}=\frac{1}{2}\left(A_{+1}^{2}+A_{-1}^{2}\right)+A_{+1}A_{-1}\cos\left(\frac{4\pi }{g}\Delta x\right)$$

The normalized AC component *S_x_* of *I_x_* can thus be obtained as follows:

(5) $$S_{x}=\cos\left(\frac{4\pi }{g}\Delta x\right)$$

Finally, the *X*-directional displacement Δ*x* can be obtained as follows:

(6) $$\Delta x=\left(\cos^{-1}S_{x}\right)\frac{g}{4\pi }$$

**Figure 3:**
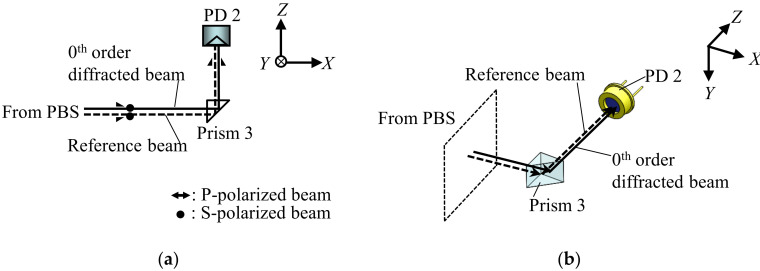
The optical design of Δ*z*-assembly: (**a**) *XZ* view; (**b**) three-dimensional view.

Similarly, Δ*z* can be obtained by the Δ*z*-assembly. Figure 3a shows the schematic of the Δ*z*-assembly extracted from Figure 1a, and Figure 3b shows its three-dimensional view. As can be seen in the figures, the zeroth-order diffracted beam from the scale grating and the reference beam reflected from the transparent grating are reflected at Prism 3 (RPB2-07-550, Sigmakoki, Japan). Then, the generated interference signal is detected by another photodiode (PD2, S1336-44BQ, Hamamatsu Photonics, Shizuoka, Japan).

Now, the electric fields of the zeroth-order diffracted beam from the scale grating and the reference beams *E*_0_ and *E*_ref_, respectively, can be expressed as follows:

(7) $$E_{0}=A_{0}\exp\left(i\varphi_{0}\right)$$

(8) $$E_{\mathrm{ref}}=A_{\mathrm{ref}}$$

where *A*_0_ and *A*_ref_ are the complex amplitude, and *φ*_0_ is the phase shift of *E*_0_ that can be expressed as follows:

(9) $$\varphi_{0}=4\pi \frac{\Delta z}{\lambda }$$

As can be seen in Equations (7) and (8), *E*_0_ will be affected by the *Z*-directional displacement of the scale grating, while *E*_ref_ will not be affected. The electric field of the interference beam *E_z_*, which is generated by superimposing the zeroth-order diffracted beam from the scale grating and the reference beam, can be expressed as follows:

(10) $$E_{z}=E_{0}+E_{\mathrm{ref}}$$

Then, the intensity of the interference signal *I_z_* can be obtained by:

(11) $$I_{z}=\frac{1}{2}E_{z}\cdot \bar{E_{z}}=\frac{1}{2}\left(A_{0}^{2}+A_{\mathrm{ref}}^{2}\right)+A_{0}A_{\mathrm{ref}}\cos\left(\frac{4\pi }{\lambda }\Delta z\right)$$

The AC component of the interference signal *I_z_*, denoted as *S_z_*, can be expressed by the following equation:

(12) $$S_{z}=\cos\left(\frac{4\pi }{\lambda }\Delta z\right)$$

Finally, Δ*z* can be calculated as follows:

(13) $$\Delta z=\left(\cos^{-1}S_{z}\right)\frac{\lambda }{4\pi }$$

**Figure 4:**
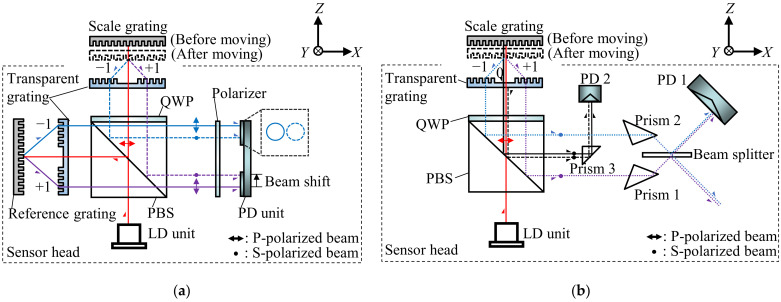
Optical configurations of the conventional surface encoder and the newly proposed surface encoder: (**a**) A schematic of the conventional surface encoder; (**b**) A schematic of the newly proposed surface encoder.

Figure 4a shows the schematic of the conventional surface encoder where the first-order diffracted beams could be shifted when the scale grating is moved along the *Z*-direction. As can be seen in Figure 4a, the first-order diffracted beams from the scale grating shift with respect to the reference beam when the scale grating travels along the *Z*-axis, making it difficult to superimpose them on the PD unit and to generate interference signals. On the contrary, in the newly proposed surface encoder shown in Figure 4b, due to the symmetrical optical configuration of the first-order diffracted beams with respect to the beam splitter, the two diffracted beams still overlap and interfere with each other on PD 1. The working distance of the sensor head can thus be expanded; namely, the *Z*-directional measuring range can be expanded. In this setup, the main factors affecting the *Z*-directional measuring range are the sizes of the transparent grating, the PBS and the prisms. Moreover, as can be seen in Figure 4a, the non-common optical paths of the measurement beams and reference beams in the conventional surface encoder are relatively long. In contrast, in the newly proposed surface encoder shown in Figure 4b, the zeroth-order diffracted beam from the scale grating has almost the same optical path as the reference beam; the non-common optical path is only twice the distance between the scale grating and the transparent grating. The reduced non-common optical path is expected to improve the measurement resolution and stability of the surface encoder.

It should be noted that in this paper, attention was paid to the proposal of the optical configuration for the surface encoder to expand its *Z*-directional measuring range. To avoid losing the focus of the study, and for the sake of simplicity, the optical components for measurement of the bi-directional displacement, which were implemented in the conventional surface encoders [25,26], have thus been omitted in the newly proposed surface encoder.

## 3. Testing of the Prototype Two-Axis Surface Encoder

**Figure 5:**
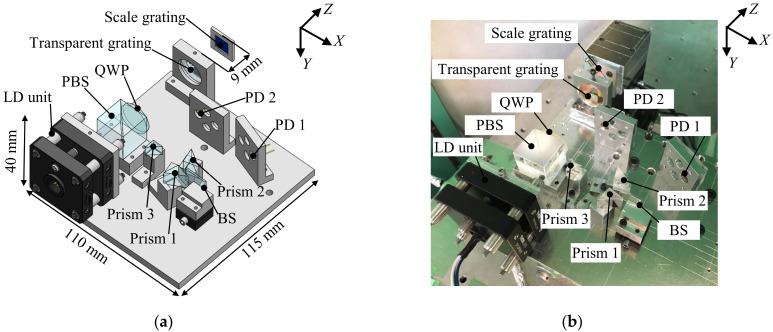
Prototype sensor head: (**a**) A three-dimensional model; (**b**) a photograph.

Figure 5a shows a schematic of the prototype sensor head for the proposed two-axis surface encoder. The size of the prototype sensor head was 110 mm (*X*) × 115 mm (*Z*) × 40 mm (*Y*). The LD unit was designed to have a laser diode with a light wavelength of 685 nm, a collimating lens with a focal length of 4.6 mm and an aperture with a diameter of 1 mm. A scale grating with a pitch of 1 µm over a length of 10 mm was fabricated based on the interference lithography, utilizing a dual-beam interferometer [25,30,31,32], and was used as the scale for the proposed surface encoder. A transparent grating fabricated using the same fabrication system as the scale grating was also employed. The initial working distance between the scale grating and the sensor head was designed to be 9 mm. Using the scale grating and the transparent grating together, the first-order diffracted beams, with an angle of diffraction of 43.24 degrees, were aligned to be parallel with the zeroth-order diffracted beam; this contributed to reducing the size of the sensor head. The beam splitter, with dimensions of 12 mm × 8 mm × 1 mm, was placed in the optical path of the first-order diffracted beams. Prisms 1 and 2, with dimensions of 10 mm × 10 mm × 10 mm, were placed symmetrically on both sides of the beam splitter. Figure 5b shows a photograph of the developed prototype sensor head.

**Figure 6:**
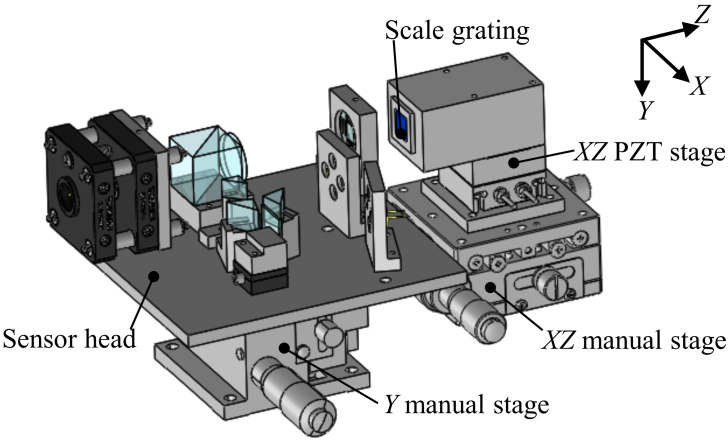
Experimental setup.

Figure 6 shows the experimental setup constructed for the evaluation of the basic performance of the developed surface encoder. A 10 mm square scale grating was mounted on an *XZ* PZT stage (P-620.2CL, Physik Instrumente, GmbH, Kirchen, Germany), which was mounted on an *XZ* manual stage (TSD-602SDM, Sigmakoki, Japan). The *XZ* PZT stage was used to generate a displacement of the scale grating with a resolution of 0.2 nm over a travel range of 50 μm along the *X*- and *Z*-directions. Because of the short travel range of the *XZ* PZT stage, an *XZ* manual stage with a travel range of ±6.5 mm was employed to move the scale grating over a long distance to evaluate the measuring range of the developed surface encoder along the *Z*-axis. The sensor head was mounted on a *Y* manual stage (LV-642-1, Chuo Precision Industrial, Tokyo, Japan), which was used for adjusting the position of the sensor head in the *Y*-direction so that the laser beam could be projected onto the middle of the scale grating.

**Figure 7:**
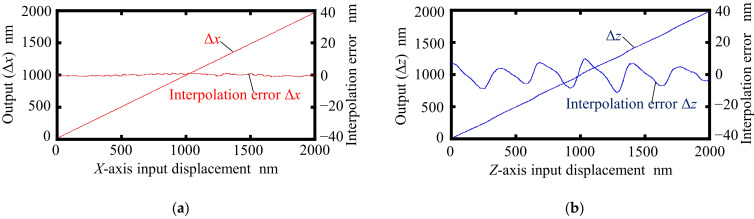
Interpolation errors: (**a**) Interpolation error in measurement of Δ*x*; (**b**) interpolation error in measurement of Δ*z*.

Experiments were carried out using the developed setup. At first, for the verification of the proposed principle for measurement of the two-axis displacements, the *X*- and *Z*-directional translational motions of the scale grating given by the *XZ* PZT stage were measured by the surface encoder. In the experiment, a displacement command was applied to the *XZ* PZT stage to move the scale grating continuously at a fixed speed of 400 nm/s along the *X*- and *Z*-directions, respectively. The displacements were calculated utilizing the interference signals obtained by the PDs. Data were collected at a sampling rate of 1 kHz. Figure 7 shows the results. The nonlinear error components are also indicated in the figure. These nonlinear errors are caused by interpolation errors, which are associated with the subdivision of the interference signal within one signal period. The peak-to-valley amplitudes of the periodic error component, corresponding to the interpolation errors, were evaluated to be approximately ±1 nm and ±15 nm in the measurements of the *X*- and *Z*-directional displacements, respectively. The periods of the observed interference signals (500 nm and 342.5 nm for the measurement of Δ*x* and Δ*z*, respectively), agreed well with those predicted from the theoretical equations. Interpolation errors can be caused by imperfections in optical elements, nonlinearities in signal processing systems such as analog circuits and AD converters, and alignment errors between the sensor head and the scale grating [33,34].

**Figure 8:**
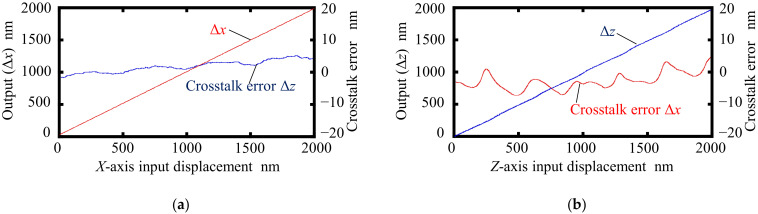
Crosstalk errors: (**a**) Readings of Δ*x* and Δ*z* when the *X*-directional displacement was applied; (**b**) Readings of Δ*x* and Δ*z* when the *Z*-directional displacement was applied.

**Figure 9:**
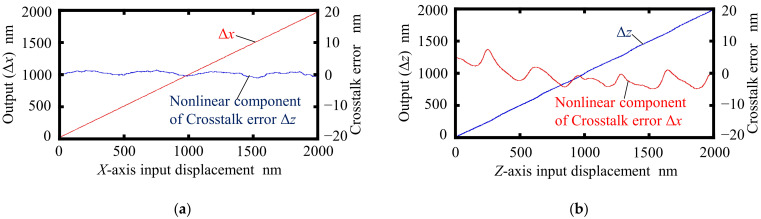
Non-linear component in the crosstalk error: (**a**) Reading of Δ*x* and the nonlinear component of the reading of Δ*z* when the *X*-directional displacement was applied; (**b**) reading of Δ*z* and the nonlinear component of the reading of Δ*x* when the *Z*-directional displacement was applied.

The crosstalk error of the prototype surface ender in each axis was investigated. Figure 8 shows the results. The interference signals *I_X_* and *I_Z_* were received simultaneously while the scale grating was made to travel along the *X*- and *Z*-directions by the *XZ* PZT stage. Figure 8a shows the variations in Δ*z* when the scale grating was moved along the *X*-direction, which indicates the crosstalk error of the prototype surface encoder with respect to the *X*-directional translational motion. Similarly, Figure 8b shows the variations in Δ*x* when the scale grating was moved along the *Z*-direction. It can be seen from Figure 8 that the crosstalk errors were approximately 13 nm and 20 nm with respect to the *X*- and *Z*-directional translational motions, respectively. Both the linear and nonlinear components were included in the crosstalk errors. The linear components of the crosstalk errors were considered to be caused by the misalignment between the axis of the sensor head and the axis of the scale grating, and the misalignment between the axis of the optical elements and the axis of the sensor head. Those linear components can be reduced by a more accurate alignment, as well as via a compensation process. Figure 9 shows the results of the nonlinear component of the crosstalk error after removing the linear component. It can be seen that the peak-to-valley amplitudes of the crosstalk error were 3 nm and 13 nm when the encoder was moved along the *X*- and *Z*-directions, respectively. Additionally, the nonlinear component of this crosstalk error showed a periodicity with respect to the movement of the scale grating. The crosstalk error component Δ*z* showed the same period as that of the *X*-directional interference signal, which was half of the grating period *g* (=500 nm). Meanwhile, the crosstalk error component Δ*x* had the same period as that of the *Z*-directional interference signal, which was half of the light wavelength *λ* (=342.5 nm).

**Figure 10:**
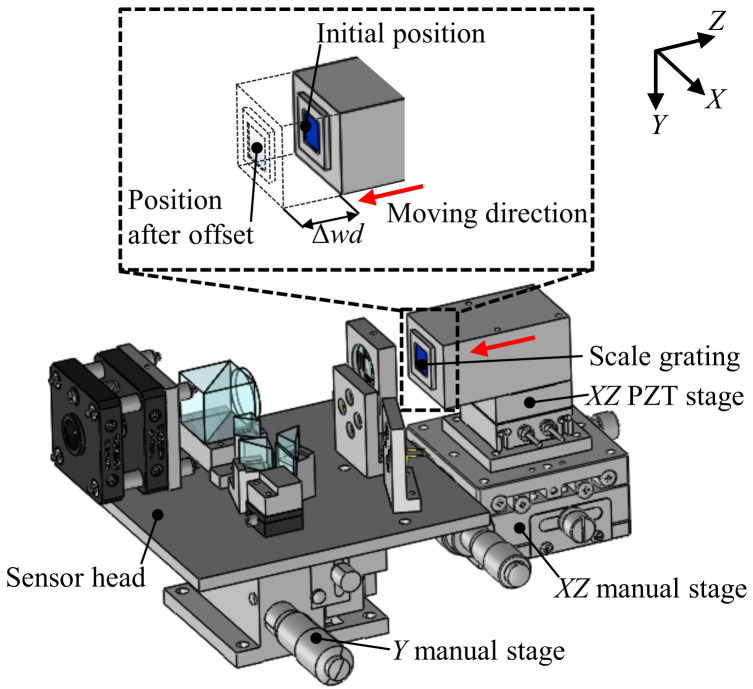
Schematic of the experimental setup for testing the *Z*-axis measuring range of the surface encoder.

Figure 10 shows a schematic of the experimental setup for testing the *Z*-directional measuring range of the surface encoder. In the experiments, the *Z*-directional offset Δ*wd* in a step of 1 mm was given to the scale grating using the *XZ* manual stage. Compensations for the offset, amplitude and phase of the interference signals were carried out for measurements at each *Z*-position of the scale grating.

**Figure 11:**
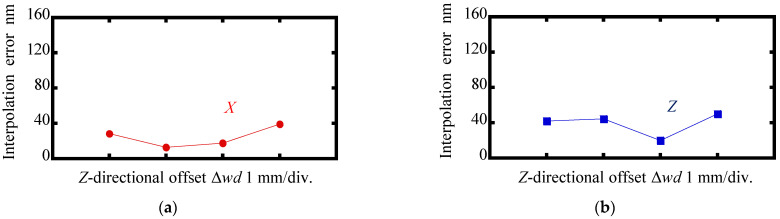
Variations in amplitudes of the interpolation errors as the change in the *Z*-directional offset Δ*wd*: (**a**) Measurement of Δ*x*; (**b**) Measurement of Δ*z*.

**Figure 12:**
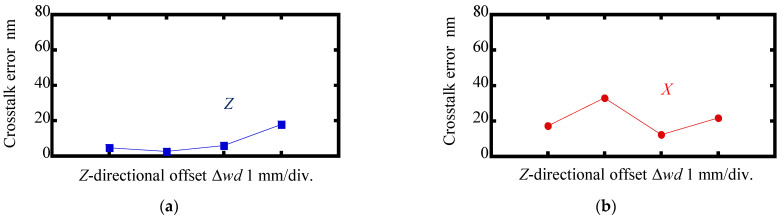
Variations in amplitudes of the crosstalk errors as the change in the *Z*-directional offset Δ*wd*: (**a**) Reading of Δ*z* when the scale was moved along the *X*-direction in a range of ±1.5 mm; (**b**) Reading of Δ*x* when the scale was moved along *Z*-direction in a range of ±1.5 mm.

Figure 11 and Figure 12 show the results. As can be seen in the figures, there were no significant changes in the amplitudes of the interpolation errors and crosstalk errors when Δ*wd* was applied to the scale grating; these results indicated that the change in the working distance of the sensor head over a range of ±1.5 mm did not affect the measurement of the *X*- and Z-directional displacements of the scale grating.

## 4. Conclusions

A new optical configuration for a surface encoder, capable of measuring the *X*-directional in-plane displacement and the *Z*-directional out-of-plane displacement of a scale grating has been proposed. To achieve a long *Z*-directional measuring range, a Fizeau-type measurement method, which uses the interference signal of the zeroth-order diffraction beam from the scale grating and the reference beam from the transparent grating, has been employed, while the *X*-directional displacement measurement has been realized through utilizing the interference signal of the positive and negative first-order diffracted beams. A prototype surface encoder, which has been designed with compact dimensions of 110 mm (*X*) × 115 mm (*Z*) × 40 mm (*Y*), has been constructed, and the feasibility of the proposed measurement method has been verified in experiments. Experimental results demonstrated that the developed surface encoder has good linearity for measurement of both the *X*- and *Z*-directional displacements, with interpolation errors of ±1 nm and ±15 nm, respectively. The peak-to-valley amplitudes of crosstalk errors were evaluated to be 13 nm and 20 nm in the *Z-* and *X*-directions, respectively. Through the experiments, it was revealed that the developed surface encoder had a *Z*-directional measuring range of ±1.5 mm, which has been expanded from that of the conventional surface encoder. It should be noted that although only the *X*-directional first-order diffracted beams were utilized in this prototype surface encoder, the *Y*-directional first-order diffracted beams are also available. They can be utilized to extend the proposed two-axis surface encoder to a three-axis surface encoder by adding a Δ*y*-assembly, which is identical to the Δ*x*-assembly, for measurement of *Y*-directional displacement. It should be noted that attention has been paid in this paper to the proposal of the new, extended *Z*-measurement mechanism in the surface encoder, and the developed prototype sensor head can carry out measurement of only uni-directional displacement of the scale grating along the *X*- and *Z*-directions. Future work will include a detailed investigation of the causes of the periodic crosstalk error components, and the modification of the optical configuration for measurement of the bi-directional displacements along the three (*X*-, *Y*- and *Z*-) axes.

## Data Availability

The data presented in this study are available on request from the corresponding author.
